# EEG desynchronization during phasic REM sleep suppresses interictal epileptic activity in humans

**DOI:** 10.1111/epi.13389

**Published:** 2016-04-25

**Authors:** Birgit Frauscher, Nicolás von Ellenrieder, François Dubeau, Jean Gotman

**Affiliations:** ^1^ Montreal Neurological Institute and HospitalMcGill UniversityMontrealQuébecCanada; ^2^ Department of Medicine and Center for Neuroscience StudiesQueen's UniversityKingstonOntarioCanada; ^3^ CONICET – LEICINational University of La PlataLa PlataArgentina

**Keywords:** Epilepsy, Intracerebral EEG, Polysomnography, High‐frequency oscillations, Sleep

## Abstract

**Objective:**

Rapid eye movement (REM) sleep has a suppressing effect on epileptic activity. This effect might be directly related to neuronal desynchronization mediated by cholinergic neurotransmission. We investigated whether interictal epileptiform discharges (IEDs) and high frequency oscillations—a biomarker of the epileptogenic zone—are evenly distributed across phasic and tonic REM sleep. We hypothesized that IEDs are more suppressed during phasic REM sleep because of additional cholinergic drive.

**Methods:**

Twelve patients underwent polysomnography during long‐term combined scalp‐intracerebral electroencephalography (EEG) recording. After sleep staging in the scalp EEG, we identified segments of REM sleep with rapid eye movements (phasic REM) and segments of REM sleep without rapid eye movements (tonic REM). In the intracerebral EEG, we computed the power in frequencies <30 Hz and from 30 to 500 Hz, and marked IEDs, ripples (>80 Hz) and fast ripples (>250 Hz). We grouped the intracerebral channels into channels in the seizure‐onset zone (SOZ), the exclusively irritative zone (EIZ), and the normal zone (NoZ).

**Results:**

Power in frequencies <30 Hz was lower during phasic than tonic REM sleep (p < 0.001), most likely reflecting increased desynchronization. IEDs, ripples and fast ripples, were less frequent during phasic than tonic REM sleep (phasic REM sleep: 39% of spikes, 35% of ripples, 18% of fast ripples, tonic REM sleep: 61% of spikes, 65% of ripples, 82% of fast ripples; p < 0.001). In contrast to ripples in the epileptogenic zone, physiologic ripples were more abundant during phasic REM sleep (phasic REM sleep: 73% in NoZ, 30% in EIZ, 28% in SOZ, tonic REM sleep: 27% in NoZ, 70% in EIZ, 72% in SOZ; p < 0.001).

**Significance:**

Phasic REM sleep has an enhanced suppressive effect on IEDs, corroborating the role of EEG desynchronization in the suppression of interictal epileptic activity. In contrast, physiologic ripples were increased during phasic REM sleep, possibly reflecting REM‐related memory consolidation and dreaming.

**Key Points**

- Interictal epileptic activity is more decreased during phasic than tonic REM sleep
- This is likely explained by EEG desynchronization, as reflected by a lower power in frequencies <30 Hz in phasic versus tonic REM sleep
- In contrast to pathologic ripples, physiologic ripples are increased during phasic compared to tonic REM sleep
- Coupling with the different REM substates might allow distinguishing pathologic from physiologic ripples

It is well demonstrated that interictal epileptiform discharges (IEDs) in focal epilepsy are influenced by sleep; IEDs are increased and more widespread during non–rapid eye movement (NREM) sleep compared to wakefulness, but they are decreased and more focal during rapid eye movement (REM) sleep.^1^, ^2^, ^3^, ^4^, ^5^, ^6^, ^7^, ^8^ We recently demonstrated that this activation of IEDs is not uniformly distributed across NREM sleep: it is enhanced by high amplitude widespread slow waves with a positive correlation between the amplitude of the slow waves and the amount of IEDs. Moreover, IEDs occurred at the transition from the activated to the deactivated state, which underscores the facilitating role of neuronal synchronization for the generation of interictal epileptic activity.^9^

For the majority of patients, IEDs occur least frequently in REM sleep.^10^ This decrease is thought to be due to electroencephalography (EEG) desynchronization mainly mediated by cholinergic neurotransmission. In the feline epilepsy model, atropine, an agent with anticholinergic properties, was shown to abolish EEG desynchronization during REM sleep and to result in an increase of IEDs and seizures.^11^

Two substates of REM sleep can be differentiated: phasic REM sleep, which is episodically present during REM sleep and which is characterized by REMs, muscular twitches, and ponto‐geniculo‐occipital waves, and tonic REM sleep, the preponderant substate of REM sleep characterized by muscle atonia and a reduction in EEG amplitude of particularly the lower frequencies.^12^ Experimental studies showed that the rate of cholinergic firing is even more increased in the basal forebrain and the reticular formation during phasic compared to tonic REM sleep.^13^, ^14^ Evidence for increased cholinergic neurotransmission in phasic REM sleep exists also for humans: low doses scopolamine—an anticholinergic drug—has a suppressive effect on phasic but not on tonic REM sleep, supporting the need of increased cholinergic neurotransmission for phasic REM sleep.^15^

High‐frequency oscillations (HFOs), comprising frequencies above 80 Hz, are brief and transient EEG patterns clearly standing out of the high‐pass filtered EEG background. HFOs occur in physiologic and pathologic conditions.^16^ In epilepsy, evidence suggests that HFOs are a more specific biomarker of the epileptogenic zone than IEDs.^17^ Previous studies showed that rates of HFOs are lowest during REM sleep,^18^, ^19^, ^20^ which is in line with the results on IEDs.^10^ A differentiation between phasic and tonic REM sleep has not been performed.

Rates of IEDs are considerably higher in intracerebral EEG compared to scalp EEG.^21^ Because rates of IEDs are usually lowest during REM sleep, intracerebral EEG is particularly useful to investigate IEDs in the context of REM sleep; rates of IEDs in REM as assessed with intracerebral EEG are still expected to be high enough for most patients to perform statistical comparisons, whereas rates in scalp EEG can be very low, and IEDs are often absent.^4^ We investigated the occurrence of IEDs across phasic and tonic REM sleep in patients undergoing combined scalp‐intracranial EEG recordings. We hypothesized that phasic REM sleep has an enhanced suppressive effect on IEDs compared to tonic REM sleep, presumably because of an increased cholinergic drive. We also analyzed the distribution of HFOs across phasic and tonic REM sleep.

## Materials and Methods

### Patient selection

We selected patients with pharmacoresistant focal epilepsy who underwent combined scalp‐intracerebral EEG (depth electrodes) recording leading to identification of the seizure‐onset zone (SOZ) at the Montreal Neurological Institute and Hospital between October 2013 and January 2015, and who had one night of polysomnography. Inclusion criteria for the present study were the following: (1) presence of at least 2 min of concatenated segments of rapid eye movement bursts >5 s (phasic REM sleep) and 2 min of control segments without rapid eye movements (tonic REM sleep); and (2) presence of at least five unambiguous IEDs in at least one intracerebral EEG channel during the combined phasic and tonic REM sleep segments. The arbitrary cutoff of 2 min of phasic REM sleep was chosen based on statistical considerations: rapid eye movements are present in approximately 10% of total REM time,^22^ and we were interested not only in isolated rapid eye movements, but in rapid eye movement bursts exceeding 5 s (for the definition of rapid eye movement bursts see below). Excluded were patients whose scalp EEGs contained too many IEDs or who showed widespread pathologic slowing during wakefulness, making correct sleep staging ambiguous or impossible. A further exclusion criterion was the presence of secondarily generalized seizures during the 12 h or focal seizures (symptomatic or asymptomatic) during the 6 h prior to the evaluated sleep recording.

Twenty‐one patients underwent intracerebral EEG recordings, and 12 were included in the current project according to the selection criteria. Table 1 provides information on the demographic, neuroimaging, and electroclinical findings of the patient group. Reasons for exclusion were the following: asymptomatic seizures during the night of polysomnography (n = 3), fewer than five unambiguous spikes during the concatenated REM sleep segments (n = 2), <2 min of phasic REM sleep (n = 2), technical problems during the night of recording affecting the electrooculography (EOG) signal (n = 1), and abundant IEDs in the surface EEG interfering with sleep scoring (n = 1).

**Table 1:** Demographic, neuroimaging, and electrophysiologic data of the patients

ID	Ictal and interictal scalp EEG findings	MR imaging	Implanted electrode positions	Interictal SEEG	SOZ SEEG	Surgery following SEEG	Pathology	Antiepileptic medication at time of investigation
1	L T	No lesion	L: OF, Ca, Cm, Is, Ip, Am,Hc,Hp,Tp,PHp R: Hc	L: Am, Hc, Hp, Tp, and T neocortex, Is R: Hc	L T and L perisplenial area > L I ≫> L T mesial	L ant T and Am	Unspecified gliosis	TPX (200) CBZ (600) Clo (30)
2	bil T, L > R	bil focal PNH: RF, R + L atrium, LO horn with L TPO abnormal gyration and atrophy	L: OF, Ca, Am,Hc,Hp,Fus,Cp,PC,LL R: Am,Hc	R: Am L: Am, Hc, Hp, Fus, LL, OF, CP, PC	R Am, L Fus, and L T neocortex	L O horn NH thermocoagulation	–	LEV (3,000) Clo (25)
3	L F T	No lesion	L: OF,Ca, Ia,Am,Hc,Hp,Ec,Fus,Ci	L: Hc + ant T neocortex, Am, Ia	L ant insula and ant T neocortex	No	–	CBZ (1,200)
4	L T P O	L post insula, T and inf P atrophy and gliosis	L: OF,Ia, Ip,Hp,Ci, PC,C,Os	L: Hp,PC,C,Os	L O and Hp	L O	FCD type III	LEV (3,000) PHT (350) Clo (40)
5	bil T	bil focal PNH: L + R atrium, L Fus, L + R T, L O horn	R: Ca,Hc, Hp L: OF,Ca, Am,Hc,Hp, Fus,Cp	R: F NH, T NH L: F NH, Am, Hc + neocortex, T NH, Fus NH + overlying neocortex	L T or bilateral T L > R, 1× R T mesial	L ant T	Glial neuronal heterotopia	VPA (2,250) LEV (3,000) CBZ (1,400)
6	bil T	L Hc atrophy	L: Am,Hc, Hp,Fus R: Am,Hc, Hp,Fus	L: Am,Hc, T neocortex R: Am, Hc, T neocortex	T neocortex, R > L	No	–	OXC (1,800) LEV (3,000) LTG (400)
7	bil F T	R Hc atrophy	L: OF,Ca, Cm,Am, Hc,Hp R: OF,Ca, Cm,Am,Hc,Hp	R: T, OF L: T, neocortex and Am	R: Hp, R T neocortex L: T neocortex, Am, Hc, Hp	R SeAH	Hc sclerosis	VPA (2,000) Clo (30) LEV (3,000)
8	L T	L Hc atrophy	L: Ca,Am, Hc,Hp,Fus, Ci, Cis,Oi	L: Am, Hc, Hp, Fus + T neocortex	L Hc and Fus	L SeAH	Hc sclerosis	LTG (400) CBZ (800)
9	R T P O	No lesion	R: Am,Hc, Hp,Ci,Cis, PC,Os,Oi	R: Am, Hc, Hp, Ci	R Hc and inf isthmus	R SeAH	Hc gliosis	LEV (2,000)
10	L T P O	No lesion	L: Am,Hc, Hp,Fus,Ci,LL R: Am,Hc	L: mesial + neocortical T,Fus,Ci R: mesial + neocortical T	L T‐O (post basal T and Fus, and ant lingual)	No	–	CBZ (1,200) LTG (300) Clo (20)
11	L F T	Hypothalamic hamartoma	L: OF,Ca, Am,Hc,Hp,Le	L: Le, Am, Hc, Hp + neocortical	Hypothalamic hamartoma	Hamartoma thermocoagulation	–	CBZ (1,200)
12	L T P O	L post T NH	L: NHa,NH, NHp,Ci, LL	L: NHa,NH, NHp,Ci, LL, max. neocortical overlying the NH	L post quadrant, max. neocortical	No	–	TPX (275) PHT (500) LEV (1,500) Clo (30)

OF, orbitofrontal; Am, amygdala; ant, anterior; bil, bilateral; C, cuneus; Ca, anterior cingulate gyrus; CBZ, carbamazepine; Ci, isthmus of the cingulate gyrus, Cis, superior part of the isthmus of the cingulate gyrus; Clo, clobazam; Cm, middle cingulate gyrus; Cp, posterior cingulate gyrus; Ec, entorhinal cortex; F, frontal; FCD, focal cortical dysplasia; Fus, fusiform gyrus; Hc, hippocampus; Hp, posterior portion of the hippocampus; I, insula; Ia, anterior insula; inf, inferior; Ip, posterior insula; Is, superior insula; L, left; Le, lesion; LEV, levetiracetam; LL, lingual gyrus; LTG, lamotrigine; NH, nodular heterotopia; NHa, anterior to the nodular heterotopia; NHp, posterior to the nodular heterotopia; O, occipital; Oi, inferior occipital; Os, superior occipital; OXC, oxcarbazepine; P, parietal; PC, Pre‐cuneus; PHp, parahippocampal gyrus; PHT, phenytoin; PNH, periventricular nodular heterotopia; R, right; SeAH, selective amydalo‐hippocampectomy; T, temporal; Tp, temporal pole; TPX, topiramate; VPA, valproic acid.

This study was approved by the Montreal Neurological Institute and Hospital Review Ethics Board. All patients signed an ethical board approved written informed consent prior to study participation.

### Intracerebral and scalp EEG recordings

An average of 8.6 depth electrodes (range 5–12) was implanted stereotactically using an image‐guided system. Table 1 provides the investigated cortical sites. Scalp EEG was obtained with subdermal thin wire electrodes at positions F3, F4, Fz, C3, C4, Cz, P3, P4, Pz. In the night of the sleep recording, which was at least 72 h after implantation, additional electrodes for EOG and electromyography of the chin were used. The EEG signal was high‐pass‐filtered at 0.1 Hz, low‐pass‐filtered at 500 Hz, and sampled at 2,000 Hz. EEG were recorded using the Harmonie EEG system (Stellate, Montreal, Canada).

### Scoring of sleep and selection of REM sleep segments in scalp EEG

Sleep was scored manually in 30 s epochs in the scalp EEG.^23^ Bursts of rapid eye movements with duration of >5 s (phasic REM sleep) were visually marked during the total night's REM sleep cycles in a consecutive manner. Segments with no rapid eye movements (tonic REM sleep) of the same duration were automatically placed with a random delay between 5 and 10 s after the end of each phasic REM segment during the same REM cycle (the amount of phasic REM sleep increases over the night^22^), and visually checked. Tonic REM sleep segments of the same duration were selected to perform direct comparisons of proportions of events between both substates (tonic REM sleep encompasses approximately 90% of REM sleep^22^). REMs were defined as sharp‐onset eye movements clearly standing out of the EOG background. Figure S1 provides EEG examples without IEDs representative for phasic and tonic REM sleep of patient 8.

### Grouping of intracerebral channels into three zones

We separated intracerebral EEG channels into three zones: (1) *the normal zone (NoZ)*: channels with normal EEG activity (absence of spikes and absence of nonepileptic anomaly during the complete intracranial recording, usually lasting 1–2 weeks), located in brain regions with no structural abnormalities as revealed by high resolution MRI, and outside the SOZ; (2) *the exclusively irritative zone (EIZ)*: channels inside the irritative zone (with IEDs) but outside the seizure‐onset zone, and (3) *SOZ*: channels showing the first unequivocal ictal intracranial EEG change at seizure onset. Channels displaying nonepileptic abnormalities, artifacts interfering with the identification of HFOs, or channels outside the brain were excluded. Suitable channels were selected independently by two of the authors (B.F., F.D.).

### Assessment of interictal epileptic activity in intracerebral EEG

Bipolar montages from one depth electrode contact to the neighboring contact were used. IEDs were visually identified in all intracerebral electrodes during the phasic and tonic REM sleep segments by one scorer (B.F.) who was blinded to the scalp EEG/EOG and the presence of REMs (in contrast to scalp EEG, depth electrode recordings are, apart from a few anecdotally reported exceptions, free of eye movement artifacts; see Fig. S1). We evaluated isolated spikes, polyspikes, and bursts of epileptic activity. The marking was performed in the channel showing the highest amplitude of the IEDs, as assessed in a referential montage. A maximum of five different spike sets comprising a minimum of five spikes was marked for each patient.^9^ The onset of the marked events was used for the statistical analysis in case of spikes and polyspikes; for bursts, the duration was used.

### Assessment of HFOs in intracerebral EEG

Ripples (>80 Hz) and fast ripples (>250 Hz) were visually marked—using a bipolar montage—by one scorer (B.F.) who was blinded to the scalp EEG/EOG and to the presence of REMs. This was done in two randomly chosen minutes of the concatenated REM segments with phasic REM sleep and an equal number and duration of concatenated segments of tonic REM sleep. As in our previous work,^9^, ^17^, ^19^ only clear events containing at least four consecutive oscillations were marked as HFOs.

### Computation of power spectral density

The power spectral density was computed in each phasic and tonic REM sleep segment using the Welch method (fast Fourier transform in moving 2 s rectangular window, no overlap). For each channel, a single spectrum was obtained for phasic REM sleep and another one for tonic REM sleep by averaging the power spectral density of the corresponding segments. For each channel the power during phasic and tonic REM sleep was computed for a low frequency (0.5–30 Hz) and a high frequency (30–500 Hz) band. A two‐way analysis of variance (ANOVA) was performed in each frequency band to compare the power (in logarithmic scale) in phasic and tonic segments and in the three previously defined zones. Planned follow‐up pairwise comparisons with Bonferroni correction for nine comparisons were also carried out. Comparisons between zones are done with unpaired *t*‐tests, and comparisons between phasic and tonic REM segments with paired *t*‐tests, since data pairs are available for each channels.

#### Hypothesis testing

We tested whether during phasic REM sleep, IEDs and HFOs (ripples and fast ripples) are less frequent than during tonic REM sleep. The null hypothesis was that there was no difference between the number of events in phasic and tonic REM sleep segments. The null hypothesis had a binomial distribution with parameter θ = 0.5, which is approximated by a normal distribution given the high number of events. We pooled the data from all patients, but in order to show the robustness of our findings we provide individual results. To show that the results are not driven by a few patients with a high number of events we repeated the analysis after limiting the number of events per patient.

To compare the ripple and fast ripple rates in the three different zones, the ripple/fast ripple rate was computed for each channel and then averaged among all the channels in each zone. The null hypothesis of equal rates between zones implies that the total number of ripples/fast ripples in each zone should be proportional to the number of channels in the zones. The Akaike Information Criterion was adopted to determine the statistical significance of the difference between the binomial model with a different parameter for each of the three zones and the binomial model with a single parameter. It is an alternative to ANOVA for nonnormal distributions.^24^, ^25^ Planned follow‐up pairwise comparison tests were also performed. For any pairwise comparison between two zones x and y, the null distribution for the total number of events is binomial with the parameter equal to the number of channels in zone x divided by the sum of the numbers of channels in zones x and y. The average rate is obtained by dividing the total number of events by the total observed time. The observed time is constant, with the same value for all patients, for tonic and phasic segments, and in the different zones. Again, the binomial distributions were approximated by normal distributions to compute the p‐values. Unless otherwise stated, the reported p‐values were corrected for multiple comparisons using the Bonferroni method.

## Results

### Analyzed channels and investigated time period

A total of 189.2 min of concatenated REM sleep (534 phasic segments: 23% from the first third, 31% from the second third, and 46% from the last third of the night; 534 tonic segments; 94.6 min each) was analyzed for IEDs (mean time of investigated REM sleep: 15.8 min per patient, range 4.2–28.8 min). For ripples and fast ripples, a total of 48.0 min of concatenated REM sleep was analyzed (2 min phasic, 2 min tonic of each of 12 patients). The total number of investigated channels was 744: 249 were part of the NoZ, 222 of the EIZ, and 273 of the SOZ.

### Power distribution across phasic and tonic REM sleep

**Figure 1:**
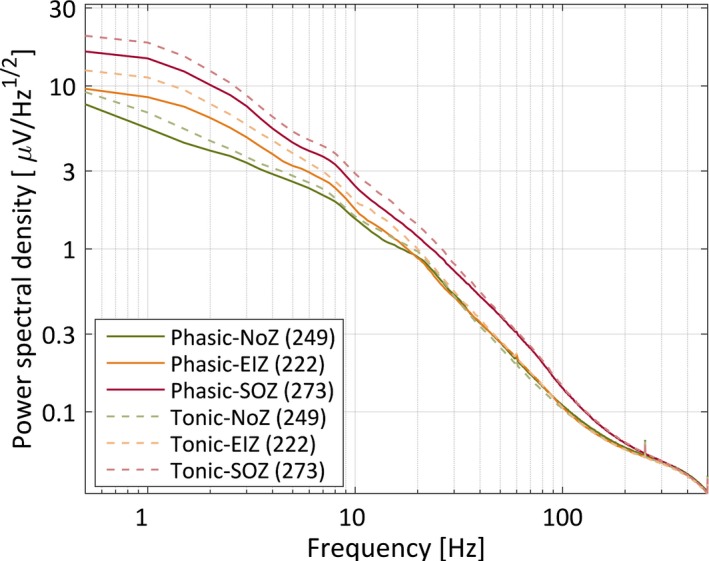
Mean power spectral density during phasic and tonic segments for channels in the normal zone (NoZ) with no epileptic or other abnormalities, in the irritative zone outside the seizure‐onset zone (exclusively irritative zone, EIZ), and in the seizure‐onset zone (SOZ); error bars were omitted for clarity.

**Figure 2:**
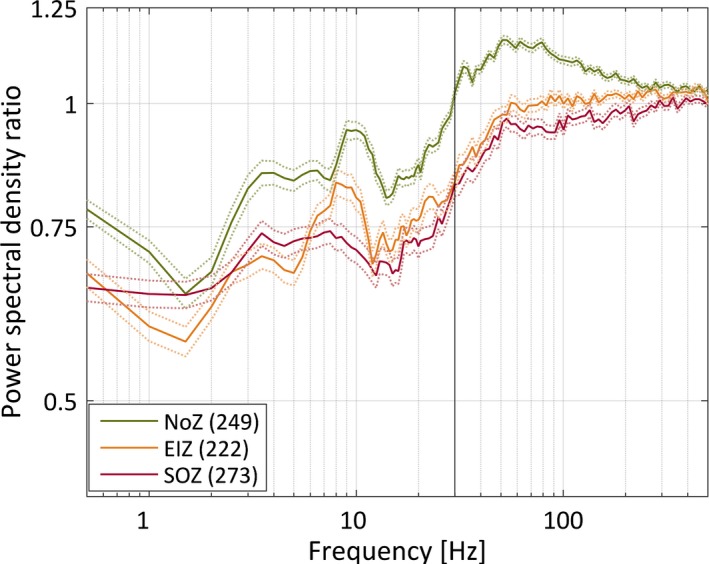
Mean ratio of the power spectral density between phasic and tonic segments for channels in the normal zone (NoZ) with no epileptic or other abnormalities, in the irritative zone outside the seizure‐onset zone (exclusively irritative zone, EIZ), and in the seizure‐onset zone (SOZ). The broken lines correspond to one standard error of the mean. The vertical line at 30 Hz indicates that this was the limit between the predefined low and high frequency bands used in the statistical analysis. In both frequency bands and for all the zones the mean power was significantly different between phasic and tonic segments (paired *t*‐tests, p‐values < 0.001); larger for phasic segments only for the high frequency band in the NoZ.

Figure 1 shows the average power spectral density during phasic and tonic REM sleep segments for the three zones. In both frequency bands (0.3–30 Hz and 30–500 Hz) two‐way ANOVAs showed significant effects of phasic and tonic REM sleep, and of the channel group. All pairwise comparisons were highly significant after correction for multiple comparisons (p < 0.001), except the difference in high‐frequency power between the NoZ and EIZ in both phasic and tonic REM sleep. Figure 2 shows the mean ratio of the power spectral density during phasic and tonic segments for the three zones, and shows that only the high‐frequency band of the NoZ has higher power in phasic than in tonic REM sleep. Because the uneven distribution of IEDs between zones could affect the results, we repeated the analysis excluding 2 s segments with IEDs. The results of the statistical tests remained unchanged.

### Distribution of IEDs and HFOs across phasic and tonic REM sleep

**Figure 3:**
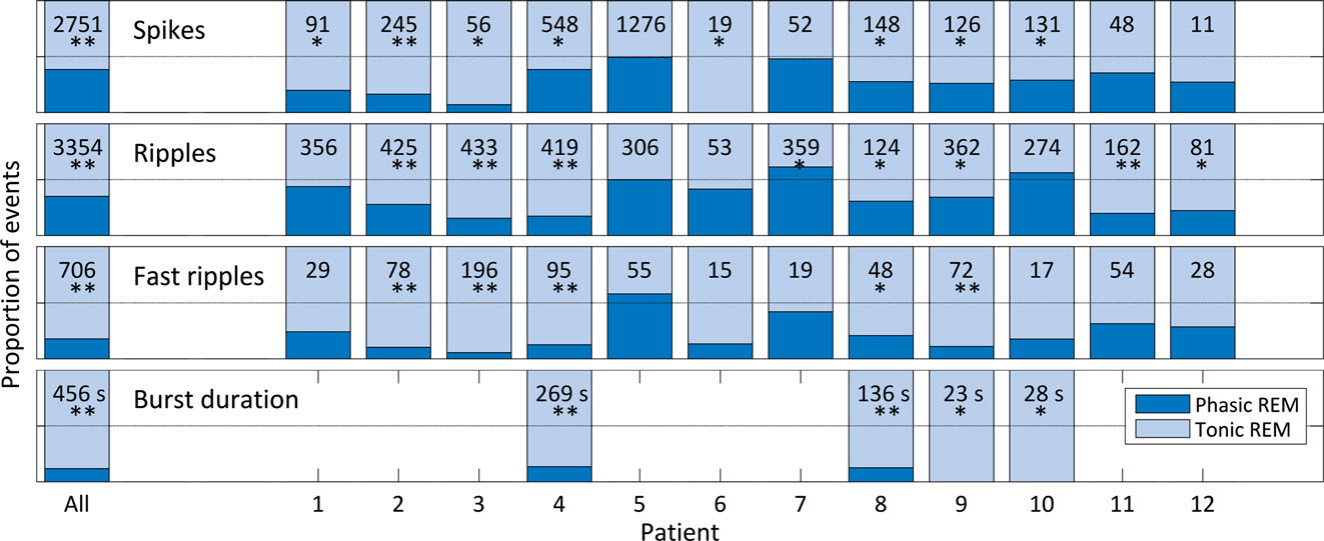
Distribution of interictal epileptic events (spikes, ripples, fast ripples, and burst duration) across phasic (blue) and tonic (light blue) REM sleep. On the left side of the panel the proportion of all events across phasic and tonic REM sleep is given; on the right side of the panel the proportions for the individual patients are given. The numbers reflect the total number of events of the categories analyzed in this study. Note that all categories of interictal epileptic events had significantly higher rates during tonic compared to phasic REM sleep. The p‐values for the individual patients are not corrected for multiple comparisons (**p < 0.001; *p < 0.05).

A total of 2,751 spikes, 3,354 ripples, and 706 fast ripples were identified. Thirty‐nine percent of spikes (1,060) occurred during phasic REM sleep compared to 61% (1,691) during tonic REM sleep (p < 0.001). On an individual level, all patients had <50% of spikes during phasic REM sleep. A significant difference was found for 8 of 12 patients (uncorrected p < 0.05). Individual results are provided in Fig. 3. To demonstrate that the results are not driven by a few patients with a high number of events, we repeated the analysis using only the first 11 spikes from each patient, that is, the minimum number among all patients. The significance of the difference between phasic and tonic REM sleep was maintained (p = 0.003) with only 24% (32 of 132) of the spikes occurring during phasic REM sleep. Bursts of spiking activity were present in 4 of the 12 patients. Total burst time during phasic REM sleep was 54 s, and during tonic REM sleep 402 s (p < 0.001). On an individual level, the four patients had significantly less burst time during phasic compared to tonic REM sleep (uncorrected p < 0.05; see Fig. 3 for individual results).

A similar distribution was found for ripples. Thirty‐five percent of ripples (1,175) were found in phasic REM sleep compared to 65% (2,179) in tonic REM sleep (p < 0.001). On an individual level, 9 of 12 patients had <50% of ripples during phasic REM sleep (uncorrected p < 0.05; see Fig. 3 for individual results). Limiting the number of events per patient to 53 (the minimum number among patients) did not alter the significance of the results (only 29% [182 of 636] of ripples occurred during phasic REM sleep, p < 0.001).

Fast ripples were even more suppressed during phasic REM sleep. Only 18% (126) occurred during phasic REM sleep, whereas 82% (580) occurred during tonic REM sleep (p < 0.001). On an individual level, 11 of 12 patients had <50% of fast ripples during phasic REM sleep compared to tonic REM sleep. This difference in distribution was significant in 5 of 12 patients (uncorrected p < 0.05; see Fig. 3 for individual results). Limiting the number of events per patient to 15 did not alter the significance of the results (only 19% [35 out of 180] of fast ripples occurred during phasic REM sleep, p < 0.001).

### Differences in ripple distribution in channels of the normal and the epileptogenic zone

The power spectral density showed an increase in the high frequency band in the NoZ (n = 249) during phasic compared to tonic REM sleep. This was not observed in the case of channels with epileptic activity (EIZ, n = 222; SOZ, n = 273; see Fig. 2). Therefore, we analyzed separately channels in the NoZ and channels with epileptic activity (EIZ, SOZ) with respect to HFO events. The statistically significant effect of including the zone as a factor in the model was confirmed by model comparison using the Akaike information criterion. The relative likelihood of the model without this factor was <0.001 for ripples and 0.016 for fast ripples.

**Figure 4:**
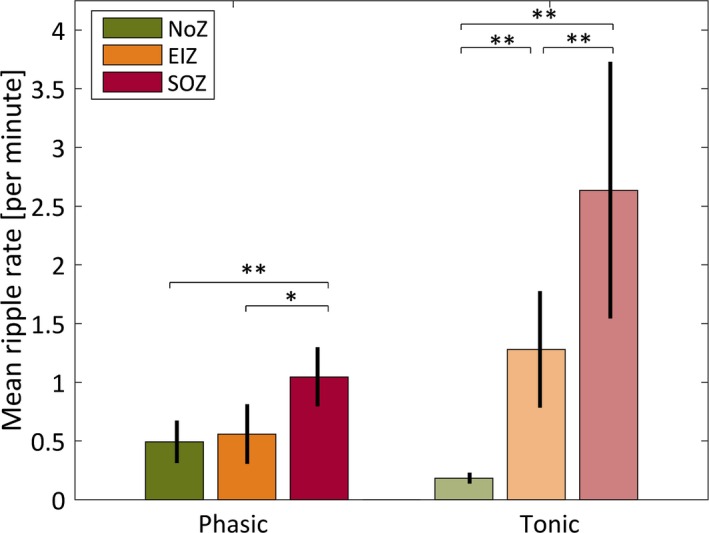
Mean ripple rates and standard errors of the mean for channels in the normal zone (NoZ) with no epileptic activity or other abnormalities, in the exclusively irritative zone (EIZ) exhibiting epileptic activity outside the seizure‐onset zone, and in the seizure‐onset zone (SOZ). The mean ripple rate in NoZ was significantly higher in phasic REM sleep compared to tonic REM sleep (p < 0.001), whereas the opposite was true for ripples in channels with epileptic activity both outside and inside the SOZ (both p‐values < 0.001). In addition, differences between zones in phasic and tonic segments were significant except between NoZ and EIZ during phasic REM sleep (*p < 0.05; **p < 0.001).

When comparing phasic and tonic REM sleep, we found that the mean ripple rate in NoZ was significantly higher in phasic REM sleep compared to tonic REM sleep (0.49/min during phasic REM sleep vs. 0.18/min during tonic REM sleep, p < 0.001), whereas the opposite was true for ripples in channels with IEDs both outside and inside the seizure onset zone (EIZ 0.57/min during phasic REM sleep vs. 1.28/min during tonic REM sleep, p < 0.001; SOZ, 1.05/min during phasic REM sleep vs. 2.64/min during tonic REM sleep, p < 0.001). Making comparisons within each state, Figure 4 shows that during phasic REM sleep the mean ripple rate in NoZ and EIZ was not significantly different (p > 0.05). There was, however, a significant difference between the mean rate in epileptic channels inside and outside the seizure‐onset zone (EIZ vs. SOZ, p = 0.004), and a highly significant difference between NoZ and SOZ (p < 0.001). During tonic segments the mean rates among the three zones were significantly different (p < 0.001). All reported p‐values are corrected to account for nine comparisons.

**Figure 5:**
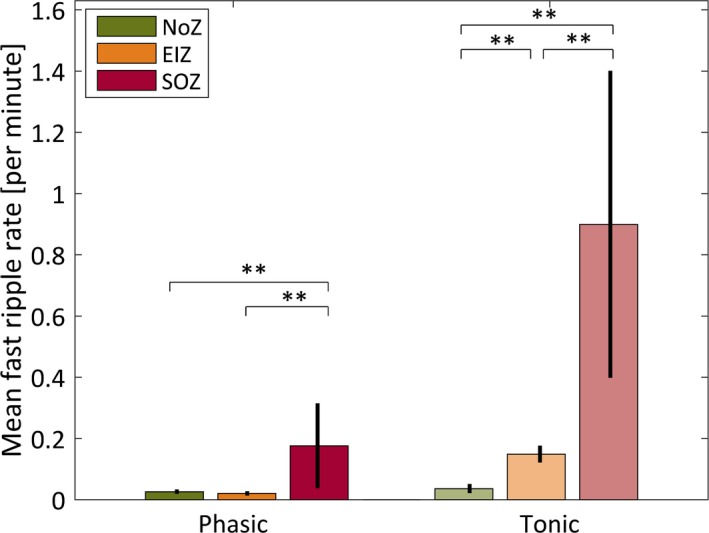
Mean fast ripple rates and standard errors of the mean for channels in the normal zone (NoZ) with no epileptic activity or other abnormalities, in the exclusively irritative zone (EIZ) exhibiting epileptic activity outside the seizure‐onset zone, and in the seizure‐onset zone (SOZ). As expected, fast ripples were sparse in the NoZ during both phasic and tonic REM sleep. For EIZ and SOZ, the differences between phasic and tonic segments were statistically significant (p < 0.001). Differences between zones in phasic and tonic segments were significant for all zones, except the rate in fast ripples in NoZ and EIZ during phasic REM sleep (**p < 0.001).

As expected, fast ripples occurred nearly exclusively in the epileptogenic zone (see Fig. 5). In line with the findings for IEDs and ripples in EIZ and SOZ, fast ripple rates were significantly less frequent during phasic compared to tonic REM sleep (EIZ, 0.02 per minute during phasic REM sleep vs. 0.15 per minute during tonic REM sleep; p < 0.001; SOZ, 0.18 per minute during phasic REM sleep vs. 0.90 per minute during tonic REM sleep; p < 0.001).

## Discussion

This study investigated the role of phasic REM sleep in the suppression of interictal epileptic activity using combined scalp and intracerebral EEG recordings. We demonstrated that (1) phasic REM sleep has an enhanced suppressive effect on IEDs and HFOs compared with tonic REM sleep, and that (2) ripples in regions with epileptic activity show a different behavior regarding the coupling to phasic versus tonic REM sleep compared to ripples in presumably normal brain regions.

### Distribution of IEDs and HFOs across phasic and tonic REM sleep

All types of IEDs were significantly less frequent during phasic REM sleep compared to tonic REM sleep. Several studies in patients with focal epilepsy have shown reduced rates of IEDs during REM sleep suggesting that REM sleep, taken globally, has antiepileptic properties.^10^ Experimentally, EEG desynchronization, present during REM sleep, was demonstrated to suppress both interictal and ictal epileptic activity.^11^, ^26^ This study showed that phasic REM sleep, where cholinergic activity is even more enhanced compare to tonic REM sleep, has more suppressive properties on IEDs and HFOs than tonic REM sleep. Cholinergic neurotransmission has a likely protective mechanism against the manifestation of epileptic activity.^27^, ^28^, ^29^ It was suggested to be further enhanced during phasic compared to tonic REM sleep.^13^, ^14^, ^15^ This additional increase in cholinergic neurotransmission during phasic REM sleep might lead to increased EEG desynchronization. Indeed, the present study corroborates this notion with a decrease in power below 30 Hz during phasic compared to tonic REM sleep.

In humans, there are two studies on IEDs pointing in this direction.^8^, ^30^ In patients with mesiotemporal lobe epilepsy, the spiking rate in foramen oval electrodes did not only outnumber the one in scalp electrodes, but also showed a different distribution across the different sleep states. In particular, phasic REM sleep exerted a significant suppressive effect on spike production compared to tonic REM sleep.^8^ This effect was confirmed with scalp EEG in patients exhibiting IEDs during REM sleep.^30^ The present study extended this knowledge by performing a direct controlled comparison of IEDs between segments with rapid eye movement bursts and segments without rapid eye movements in an unselected sample of consecutive patients with therapy‐refractory epilepsy undergoing combined scalp‐intracerebral EEG recording with IEDs occurring at different sites of the brain. Because IEDs are more frequent in intracerebral recording compared to scalp EEG recording,^21^ only two patients from the initial sample had to be excluded because of few or no IEDs during REM sleep.

### Differences in ripple rates in normal versus epileptic channels

We demonstrated that the power spectral density increased in the high‐frequency band in normal channels during phasic compared to tonic REM sleep. This was not observed in the epileptogenic zone. Moreover, we found that ripple rates behave differently in the NoZ compared to the epileptogenic zone during phasic and tonic REM sleep: in brain regions devoid of epileptic activity, ripple rates increase during phasic REM sleep, whereas in brain regions showing epileptic activity, the opposite is observed and ripple rates are higher during tonic REM sleep. We believe that this is an important observation that may help to differentiate physiologic and pathologic ripples. This finding goes well with the literature suggesting that phasic REM sleep plays an important role in learning and memory.^31^, ^32^, ^33^, ^34^ Based on the increased suppression of epileptic activity during phasic REM sleep and the fact that the amount of phasic REM sleep is higher during the later compared to the earlier REM cycles, one might further speculate that the suppression of IEDs and pathologic ripples is most pronounced during the last REM sleep cycle. This awaits future confirmation.

### Implication of the different coupling of ripples in relation to phasic and tonic REM sleep

This different coupling of ripples to phasic and tonic REM sleep suggests that the distribution pattern of ripples in relation to the state of REM sleep may help to distinguish between physiologic and pathologic ripples. This is important and could be used to improve the SOZ localization, especially in brain areas where physiologic HFOs are frequent such as in the paracentral areas, the hippocampus, and the occipital cortex.^35^, ^36^, ^37^, ^38^, ^39^ Few studies have focused on the differentiation between physiologic and pathologic HFOs, and none has yet defined a reliable marker to distinguish between both entities.^37^, ^38^

## Limitations

Although the design of our study does not allow a demonstration of causality, the most likely explanation for our findings is that EEG desynchronization suppresses interictal epileptic activity during REM sleep. We define the term EEG desynchronization as an increase of power in higher frequencies and a decrease of power in lower frequencies. This commonly used definition of EEG desynchronization does not necessarily reflect neuronal desynchronization, since neuronal synchronization could theoretically occur at low and at high frequencies. Moreover, we do not refer to phase synchrony of the EEG signals. We did not assess the IED index during NREM sleep. We can therefore not exclude that a minority of the investigated patients had in contrast to the usual presentation^1^, ^2^, ^3^, ^4^, ^5^, ^6^, ^7^, ^8^, ^10^ a higher IED index during REM compared to NREM sleep. This, however, would not impact the present findings and their interpretation. We also cannot completely rule out that the heterogeneity of the studied patients as well as the use of antiepileptic medication, which is known to have potentially altering effects on sleep,^40^ might have had an influence. The fact that suppression of epileptic activity was more pronounced during phasic than tonic REM sleep across all patients irrespective of the type of epilepsy and antiepileptic medication, does, however, rather underscore the robustness of the effect of phasic REM sleep on IED suppression. If this effect is also present for the continuous or semicontinuous IED activity present in focal cortical dysplasia remains to be seen and awaits further investigation.

## Conclusion

We showed that phasic REM sleep had an even more enhanced suppressive effect on IEDs than tonic REM sleep, underscoring the possible protective property of desynchronization in the generation of interictal epileptic activity. In contrast, physiologic ripples were enhanced during phasic REM sleep, which might reflect cognitive processes that have been suggested to take place during this substate of REM sleep. Whether the coupling of HFOs to tonic REM sleep might aid in the delineation of the SOZ awaits future confirmation.

## Disclosure of Conflict of Interest

No financial disclosures related to this project have to be disclosed. Outside of the submitted work, B.F. has received a speaker's fee from Novartis Japan, and N.v.E. and J.G. have received fees for consultancy from Precisis Inc. F.D. has nothing to disclose. We confirm that we have read the Journal's position on issues involved in ethical publication and affirm that this report is consistent with those guidelines.

## Supporting information


**Figure S1.** Representative EEG examples taken from sections without IEDs of phasic (**A**) and tonic (**B**) REM sleep of patient 8.
