# Supplementary material for: EEG desynchronization during phasic REM sleep suppresses interictal epileptic activity in humans
Source: Epilepsia. 2016 Apr 25;57(6):879–88. doi: 10.1111/epi.13389 (PMC4949560; doi:10.1111/epi.13389)
Supplement: Supplementary file 1 — Figure S1. Representative EEG examples taken from sections without IEDs of phasic (A) and tonic (B) REM sleep of patient 8. [file EPI-57-879-s001.docx]

**SUPPORTING INFORMATION**

**
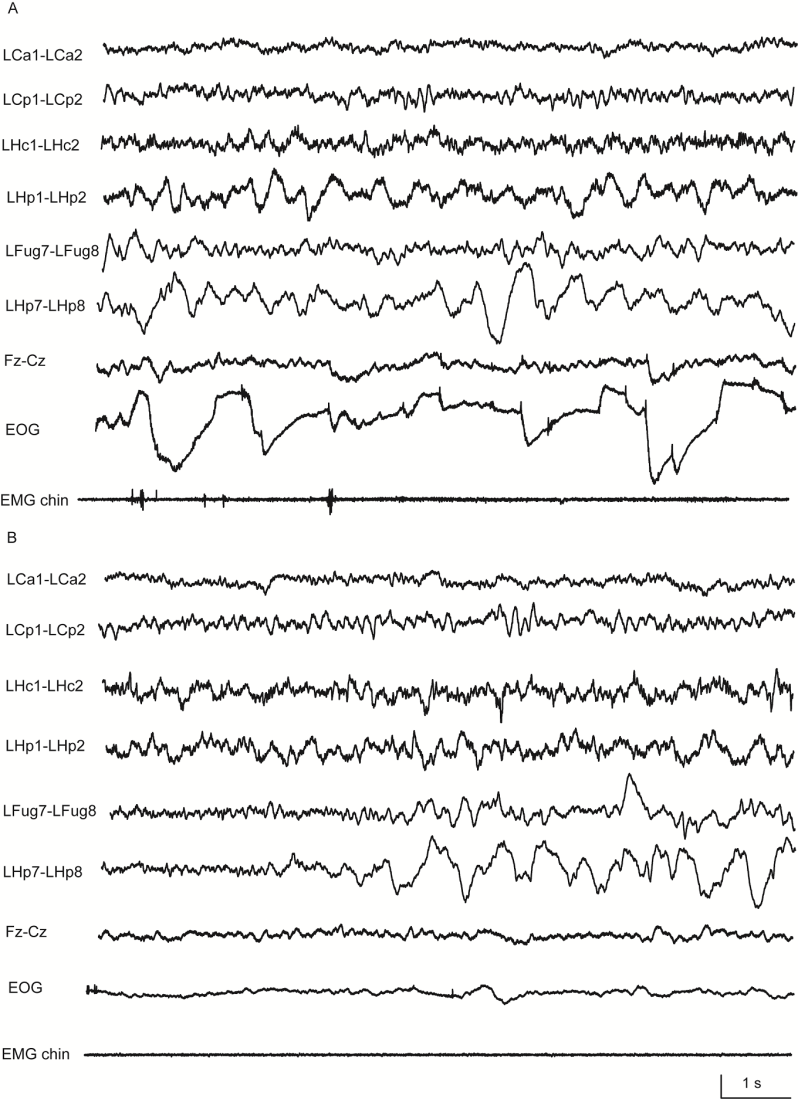
**

**Supporting Figure 1.** Representative EEG examples taken from sections without IEDs of phasic (A) and tonic (B) REM sleep of patient 8. Note that the EEG (although showing some differences across the different cortical regions) of phasic and tonic REM sleep is similar when visually analyzed. The two upper channels represent channels within the NoZ (anterior cingulate, posterior cingulate), the two middle channels channels of the SOZ (anterior and posterior portion of the hippocampus), and the two lower channels channels of the EIZ (posterior T neocortex). As expected, the SOZ cannot be identified based on this interictal EEG sample. NoZ, normal zone; EIZ, exclusively irritative zone; SOZ, seizure onset zone.
